# Recent Approaches to the Formulation, Uses, and Impact of Edible Coatings on Fresh Peach Fruit

**DOI:** 10.3390/foods13020267

**Published:** 2024-01-15

**Authors:** Muhammad Aaqil, Chunxiu Peng, Ayesha Kamal, Taufiq Nawaz, Jiashun Gong

**Affiliations:** 1 College of Food Science and Technology, Yunnan Agricultural University, Kunming 650201, China; aaqilkhan26@gmail.com; 2 College of Horticulture and Landscape, Yunnan Agricultural University, Kunming 650201, China; pengchunxiu2023@163.com (C.P.); ayeshakamal235@gmail.com (A.K.); 3 College of Natural Sciences, South Dakota State University, Brookings, SD 57007, USA; taufiqnawaz18@gmail.com; 4 Agro-Products Processing Research Institute, Yunnan Academy of Agricultural Sciences, Kunming 650221, China

**Keywords:** peach fruit, edible coating, shelf life, hydrocolloids, lipids, postharvest storage

## Abstract

Peaches are among the most well-known fruits in the world due to their appealing taste and high nutritional value. Peach fruit, on the other hand, has a variety of postharvest quality issues like chilling injury symptoms, internal breakdown, weight loss, decay, shriveling, and over-ripeness, which makes a challenging environment for industries and researchers to develop sophisticated strategies for fruit quality preservation and extending shelf life. All over the world, consumers prefer excellent-quality, high-nutritional-value, and long-lasting fresh fruits that are free of chemicals. An eco-friendly solution to this issue is the coating and filming of fresh produce with natural edible materials. The edible coating utilization eliminates the adulteration risk, presents fruit hygienically, and improves aesthetics. Coatings are used in a way that combines food chemistry and preservation technology. This review, therefore, examines a variety of natural coatings (proteins, lipids, polysaccharides, and composite) and their effects on the quality aspects of fresh peach fruit, as well as their advantages and mode of action. From this useful information, the processors could benefit in choosing the suitable edible coating material for a variety of fresh peach fruits and their application on a commercial scale. In addition, prospects of the application of natural coatings on peach fruit and gaps observed in the literature are identified.

## 1. Introduction

Peach (*Prunus persica* L.) is a tropical stone fruit from the Rosacea family cultivated throughout the world. It is assumed that in 1000 B.C., the fruit had originated from China. Across Europe, the peaches were grown by Romans, who later spread them to the rest of the world [1]. The Food and Agriculture Organization (FAO) [2] declared that about 22.8 million tons raised in peach and nectarine production were found across the globe, of which 9.9% were produced in the U.S., 19.8% were produced in Europe, and 66.1% were produced in Asia. China is the leading producer, with a production of 57.9%, having an area of 51.1%. Peach fruit is also termed a drupe (simple fleshy fruit that usually contains a single seed). The structure of the fruit consists of a delicate exocarp, a flesh or pulp that is juicy and fleshy called a mesocarp, and a hard pit called an endocarp. Color, flavor, texture, aesthetic, nutritional value, aroma, and food safety are important quality parameters for peach fruit marketability (Table 1). Peaches are considered highly valued fruits because of their delicious taste and flavor [3]. Peach fruit is regarded as one of the most valuable fruits because of its appealing taste and high nutritional value. Peach fruit’s major constituents include 86.9 g water, 11.4 g carbohydrates, and 0.7 g and 0.2 g protein and fats, respectively. It is also composed of 0.02 mg thiamine, 0.04 mg riboflavin, 7 mg calcium, 0.1 mg zinc, 0.7 mg niacin, 24 mg phosphorous, 11 mg iron, 0.9 g fiber, 9 mg ascorbic acid, and an edible part that contains 59 mg β-carotene per 100 g [4].

### Postharvest Complications

**Figure 1:**
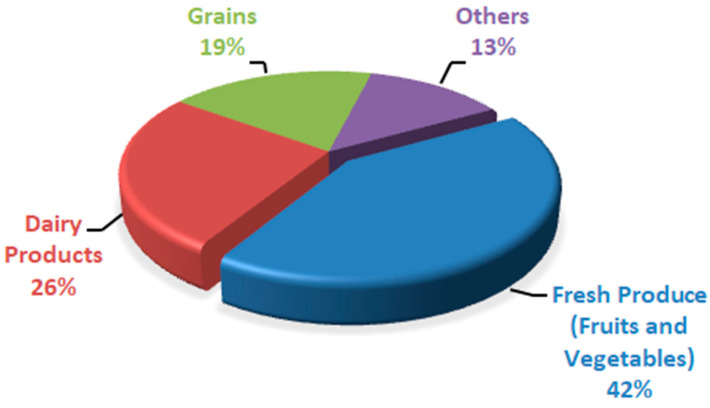
Quickly spoiled food products expressed in percentage.

Peach fruit has a high respiration rate and quick ripening process because of its climacteric nature, so it has a shorter shelf life at ambient temperature. The main reasons associated with postharvest life reduction include firmness loss, reduction in fruit quality, and decay. With time, the quality of fruit is reduced because of postharvest factors such as softening of fruit and rot development; that is why peaches are marketed soon after harvesting [5]. Because of high respiration, peach fruit has a fast ripening process, so it cannot be stored for a long period at ambient temperature. It has a shorter storage life of 3–5 days at room temperature [6]. There are approximately 40% postharvest losses in peach fruit [7]. As a result, huge financial losses are faced because there are abundant peaches in the market during peak season, and a huge portion goes wasted. At ambient temperature, peach fruit quickly ripens and spoil, so storing peach fruit at a cold storage of 0 °C results in the chilling injury of peach fruit; once the fruit moves from cold storage to room temperature, it looks normal but does not ripen well, which results in quality losses such as dry, mealy texture, tissue browning, failure to ripen, abnormal flesh color, and loss of flavor [8,9,10]. Peaches also have dynamic living tissues like other fruits, so they have a shorter shelf life and are highly perishable [11]. Physiological disorders, metabolic changes, decay, mechanical damage, and reduced firmness in stored peaches result in postharvest quality loss of peach fruit. Several factors affect these losses, such as handling, storage conditions, and ripeness stage at harvest time [12]. Without any treatment or low temperature, the storage life of peaches is much shorter at ambient temperature, which results in color and texture degradation of peach fruits during the storage period [13]. There are about 40–50% postharvest losses of peaches. In third-world nations, this value rises due to a lack of postharvest handling facilities [14]. According to Sridhar et al. [15], the wastage of different food commodities was expressed in percentages based on rapid spoilage (Figure 1). Fresh produce (fruits and vegetables) was found to be a less expensive and quickly spoiling commodity, followed by dairy items. Postharvest diseases result in around 20% of peach fruit losses because of Rhizopus rot and brown rot caused by Rhizopus stolonifera and Molinia fructi cola, respectively [16]. Botrytis cinerea results in grey mold rot in peach fruit during postharvest storage [17]. Fruit storage life is extended through low-temperature storage or shipping. Still, fruit from some cultivars results in flesh translucency, flesh browning, red pigment (bleeding), black pit cavity, failure to ripen, lack of juiciness/mealiness/wooliness, and loss of flavor after ripening at room temperature or after long-term cold storage [18]. In developing countries, the world population is increasing at a high rate, and those nations are already facing food safety and food security issues. It is a big challenge for mankind to meet the food requirements of an increasing population. By 2050, it is expected that the population across the globe will exceed 9.1 billion inhabitants, which requires about a 70% increase in fresh produce to meet the needs [19]. Excluding some plum cultivars like “Sweet Miriam” and cherries, stone fruits are considered climacteric [20]. During the ripening process, ethylene biosynthesis, which accelerates fruit enzymatic and biochemical reactions, was observed in climacteric fruits [21]. For those reactions, oxygen serves as substrate; that is why, with a rise in ethylene production, the respiration rate also rises [22]. During respiration, carbohydrates and other substrates like proteins, organic acids, and fats are metabolized. After metabolism, these substances cannot be replenished once the fruit or vegetable is cut off from the plant [23]; with the passage of time, the quality of food results in deterioration in terms of color, flavor, weight, and nutritional value. Water loss is the major factor that plays a vital role in quality deterioration in fruits and vegetables, leading to wilting, shriveling, texture loss, flaccidness, and loss of nutritional value [24]. Over the past few decades, fresh agricultural products and processed foods have benefited greatly from the widespread use of conventional petroleum-based plastic packaging. This is primarily due to the manufacturing simplicity, cheaper cost, ease of use, and favorable physico-chemical properties of the material [25]. Still, as public awareness grows related to the harmful effects of plastic on the environment, consumers are diverted toward biodegradable, renewable, and environmentally friendly packaging material [26]. Using edible films and coating is one of the ways to meet the current demand for coatings [27].

On fruit surfaces, the edible coating acts as a barrier to moisture or gas, reducing moisture loss and oxygen uptake from the surroundings, which slows down the respiration process. Food additives like antimicrobials and antioxidants were also added to the coating, so the layer acts as a career for additives that retards decay without affecting food quality. It is almost a century-old technique to use edible coatings on fruits. Wax was used for lemons and oranges in the early 12th century to maintain quality for a prolonged time [28], so for a long period of storage, the best postharvest practices are required to preserve the quality of a product.

Numerous reviews emphasize the widespread use of natural edible films and coatings for fresh fruits and vegetables [29,30]. As per our information, despite shriveling, moisture loss, and the highly perishable nature of peach fruit, no review was carried out on edible coating application on peach fruit to control decay and physiological disorders in peach fruit; this study concentrates on the most recent findings of the postharvest application of edible coatings to extend storage life and maintain quality.

## 2. Natural Edible Coatings: An Overview

**Figure 2:**
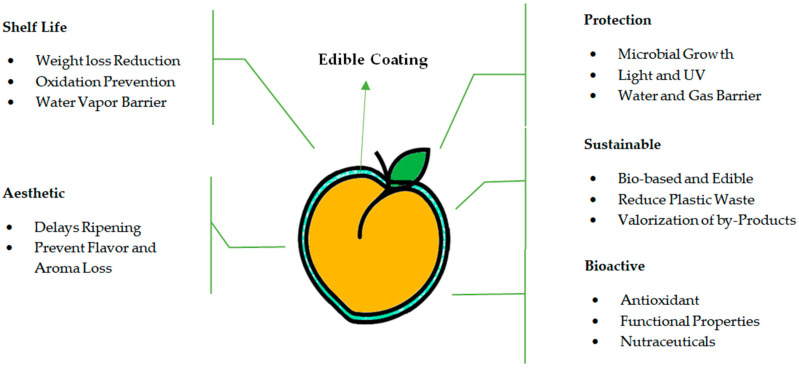
Functional properties of edible coatings applied on fresh peach fruit.

The term “edible coating” refers to a delicate fine layer of natural edible films that covers the outer part of products as an alternative to natural waxy coating. These edible material coatings reduce the risk of decay without affecting the quality of the product and prolong the storage life of fresh fruits without anaerobic conditions. These coatings preserve freshness, aroma, texture, flavor, and nutritional quality, allowing the gaseous exchange [28]. For storing postharvest agricultural products, edible coating technology has been used extensively recently. Recent studies revealed that it improves the quality of postharvest produce and thereby affects the redox state and rate of respiration [31]. Edible coatings of food-grade materials are also fit for human consumption [26]. Edible coatings block improving physical, chemical, and biological factors [32]. Biodegradable or edible coatings must possess important functional properties like a solute or gas barrier, a moisture barrier, lipid/water solubility, mechanical characteristics, nontoxicity, and color/appearance (Figure 2). The impact of edible coatings on fresh produce greatly depends on alkalinity, coating thickness, temperature, type of coating, condition, and variety of fresh produce [33]. Consumers target the wholesomeness and quality of fresh produce while purchasing based on the aesthetic of fresh produce. The major problem faced by the fruit industry is to control and maintain fresh produce quality, retard the growth of pathogenic microorganisms, and avoid spoilage [34]. To prolong the postharvest storage life of fresh-cut fruits and vegetables, edible coating application is considered a packaging strategy. Natural resources originating from edible coating are considered eco-friendly and can enhance fresh produce quality [35]. Using edible coatings as a postharvest technology was found to reduce quality losses in peaches [22]. The best coatings can reduce decay, have no adverse effect on the quality of fresh produce, and can extend storage life without causing anaerobiosis. In the past, edible coatings were used to reduce moisture loss; however, novelties in edible coating formulations possess a broad range of permeability characteristics that extend the application of edible for fresh produce [36].

Natural coatings are applied by different means on the surface of fresh produce, such as spraying, dipping, and brushing, to alter the atmosphere within the fruit [37,38,39]. Material used in films and coatings preparation should be Generally Recognized as Safe (GRAS) because it should be consumed. The fruit coating concept originated in China in the 12th century, where lemons and oranges were brought for waxing to inhibit loss of moisture and enhance aesthetic value [40], but the commercial application of wax to inhibit postharvest losses of fresh produce did not begin until 1922 [41]. However, the use of those waxes was considered unfit for consumers, paving the way for a coating application that is more sustainable and natural. An edible coating reduces quality losses by forming a semipermeable barrier around the fruit’s surface that regulates gaseous, moisture, and solute exchanges in the fruit and external surroundings [42]. A fruit’s epicarp is completely covered by edible coating; it also seals stomata and lenticels and covers pores and cracks [43]. Therefore, the coating may delay physicochemical changes, prevent physiological disorders, and slow down ripening [44]. Researchers in the field have recently paid a lot of attention to creating and using agricultural bio-based polymers like cellulose derivatives, starches, chitin/chitosan, proteins, lipids, and gums [45].

## 3. The Need for a Natural Edible Coating

The barrier that edible films and coatings create against gases and moisture modifies the atmosphere inside fresh produce; as a result, shelf life is prolonged, and the freshness of the product is maintained. It has been concluded from various studies that an edible coating alters the atmosphere inside fruit; as a result, a reduction in postharvest losses was found in peaches (Table 2). An edible coating contributes toward hygiene and acts as a barrier against microbial invasion.

Anti-browning agents, nutrients, colorants, flavors, and spices are some additives that can be added to the polymer matrix and eaten with the fruits; they also improve safety or even fruits’ sensory and nutritional qualities. Because of their biodegradable nature, edible coatings help deplete the waste produced by synthetic packaging [28].

## 4. Classifications of Edible Coatings for Postharvest Application of Peach Fruit

**Figure 3:**
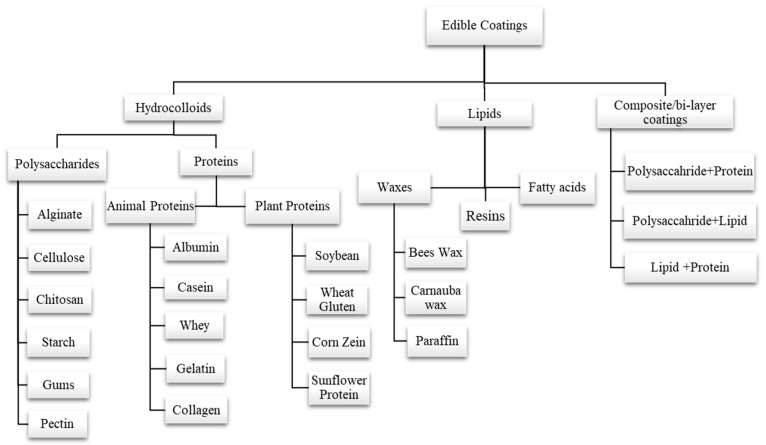
Classification of various edible coatings into different types on the basis of formulation.

The formulation of edible coatings can be made from different materials with desired properties. Polysaccharides (pectin, starch, gums, alginate, chitosan, cellulose, etc.), proteins (gelatin, egg albumin, wheat gluten, zein, whey protein, casein, soy protein, etc.), and lipid compounds (fatty acids, waxes, etc.) are three major categories that can be considered to classify the basic materials that are used to make edible coatings. Another coating is called composite coating, which results from combining more than one substance or material [57,58,59,60]. Classification of various edible coatings is depicted in (Figure 3). Edible coatings also enhance functional properties by incorporating antioxidants, antimicrobial compounds, vitamins, and minerals into a polymer matrix [61]. 

### 4.1. Lipids

The lipid-based edible coating has low water affinity as it is hydrophobic and has excellent moisture barrier properties [42]. However, it has been reported that lipid-based edible coatings have poor gas barriers and mechanical properties [62]. Apart from preventing water loss, lipid-based edible coatings reduce respiration rate, prolong postharvest life, and enhance the aesthetic of fresh produce by imparting shine on the surface [28]. Coatings made of lipids have been used for more than 800 years. In the past, coatings made of lipids were used to coat confectionery items and waxing fruits. Beeswax and paraffin wax were considered the most effective. Lipids prevent the chilling injuries that often occur in cold storage [63]. In various edible coatings, carnauba wax and beeswax (natural waxes) have been utilized as lipid components [64]. Because of their hydrophobic nature, these compounds help to prevent weight loss caused by dehydration during storage and impart gloss to the fruit’s surface. Compared to protein and polysaccharide coating, a lipid-based coating has excellent barrier properties and the best compatibility with other coating agents. The lipid-based coating leads to unacceptable organoleptic properties because of its greasy nature and lipid rancidity [65]. Widely used materials for coatings include beeswax, acetylated monoglycerides, carnauba wax, mineral oil, vegetable oil, surfactants, and paraffin wax [63]. 

### 4.2. Proteins

Proteins are classified into two groups based on the source from which they are obtained, i.e., globular protein and fibrous protein. Globular proteins are attained from plant sources (e.g., cotton seed protein, wheat gluten, cotton seed protein, corn-zein, peanut protein, soy protein) and are soluble in bases, salts, and aqueous solutions of acids or water, while fibrous proteins are attained from animal sources (e.g., collagen, whey protein, keratin, gelatin, casein) and are insoluble in water [66]. The coatings obtained from animal sources (such as milk protein) and plant sources (such as zein, wheat gluten, and soy protein) have excellent lipid, oxygen, and carbon dioxide barrier properties, especially at low RH [67]. Edible coatings from protein sources possess excellent gas barrier and mechanical properties, but their use is limited due to allergenic risks and ethical or religious beliefs [62]. Additionally, edible coatings obtained from protein sources were susceptible to cracking and brittle [65]. Various fruits and vegetables can be coated with proteins based on edible coatings derived from gelatin, milk, corn, soybeans, wheat, peanuts, or gelatin. Most protein-based films have good results on hydrophilic surfaces, but they rarely resist water vapor diffusion. Coatings derived from proteins have poor mechanical and water barrier properties but excellent oxygen and carbon dioxide barrier properties [68]. Compared to polysaccharide films, the protein coating has good mechanical and excellent gas barrier properties. Still, as it is hydrophile, like polysaccharide films, the protein coating is considered to have a poor moisture barrier [69]. It has been assumed that it has a great potential for aromatic and organoleptic attribute retention, develops a barrier against mechanical strength, and has a high oxygen permeability, but because of its hydrophilic nature, it is not a good moisture barrier that can be strengthened by the addition of hydrophobic substance like lipids [70]. 

### 4.3. Polysaccharides

Polysaccharides are biodegradable macromolecules that are safe, non-toxic, and highly stable [28]. Polysaccharides are naturally occurring polymers used in the production of edible coatings. Chitosan, starch, and gums are important ingredients used for food preservation in polysaccharide-based edible natural coatings. This coating has many advantages, like easy availability and cheap cost. However, different polysaccharides were found to have a lower water barrier quality. Some polysaccharides like carrageenan and alginate are thick-filmed and have a hygroscopic nature. Polysaccharide-based edible coatings possess antimicrobial and antioxidant characteristics, which are considered efficient in keeping fruits and vegetables fresh and improving quality. Because of its hydrophilic nature, it cannot act as a barrier to moisture [71]. Throughout the literature, polysaccharides, such as starches, gums, cellulose derivatives, and pectins, are widely mentioned and highly favored for use as an edible coating in all types of stone fruit [72]. Polysaccharides are excellent coating materials because of their easy availability, allergen-free nature, and typically water-soluble nature. Their strong hydrogen-bonded and orderly network structure leads to a good gas barrier and mechanical properties [62]. A variety of polysaccharides can be utilized in the production of edible coating. The most often available polymers are extracted from agricultural plants, animals, and marine sources. During short-term storage, these coatings have been used to prevent some food moisture loss [63].

### 4.4. Composites or Bi-Layer Natural Edible Coatings

For improved functional qualities in coatings, the development of bi-layer and composite coatings containing proteins, lipids, and polysaccharides has received much attention in the past few years. Multiple coating materials are combined in a composite edible coating, resulting in the improved functionality of the coating due to several advantageous properties [62]. Because each coating material serves a distinct but limited function, the functionality can be enhanced by combining two distinct coating materials. The main goal of composites is to maximize the benefits of the mixture’s highest possible performance while maintaining the quality as stable as an individual component. Combining multiple materials can create composite edible coatings with distinctive properties [70]. Multi-component or composite films possess both features of hydrocolloid (proteins or polysaccharides) and lipid components; hydrocolloid serves as a selective barrier to oxygen and carbon dioxide, while lipid components serve as an excellent barrier to water vapor [73]. A coating composed of just one material, such as a protein, polysaccharide, or lipid, may be effective in exhibiting a specific property but may not possess several properties or barriers simultaneously. For example, hydrocolloids (proteins and polysaccharides) are excellent film-forming materials with good structural and mechanical properties. Still, they often have a poor moisture barrier, so to enhance the hydrophobicity, lipids are often added to the edible coating to create a barrier to postharvest moisture loss. As a result, the hydrophobic nature of the lipids may play a significant role by acting as an excellent barrier to water. In other words, bi-layer or composite coatings are designed to integrate the benefits of lipids and hydrocolloid coatings to reduce or mask each limitation [68]. Edible coatings comprised of protein, polysaccharides, and lipid blends may vary in nature. This strategy assists an individual in taking advantage of the different functional properties of each film class [74].

## 5. Edible Coatings’ Effects on Physico-Chemical Properties of Peach Fruit

### 5.1. Pigments and Color Attributes

Color is a crucial sign of ripening, which determines consumer acceptability and fruit quality. Chlorophyll is broken down as the fruit ripens, and other pigments like anthocyanins and lycopene develop [75]. When compared to uncoated fruits, edible coatings retard color change and slow down ripening [76]. Depending on stone fruit types, visible color changes may occur during the ripening. Stone fruit undergoes color changes throughout maturation and ripening due to the synthesis of carotenoids and anthocyanins [77]. It has been concluded that a reduction in color changes was found in peach fruits coated with edible coatings [22,55]. Edible coatings lower the activities of a pair of important enzymes involved in the synthesis of anthocyanin, flavanone synthase, and phenylalanine ammonia-lyase, as respiration is repressed by edible coatings, which, in turn, decreases the availability of oxygen within the fruit [44]. Carotenoid synthesis was catalyzed by two enzymes, f-carotene desaturase and phytoene desaturase/synthase [78]. Peach fruits coated with aloe arborescens and aloe vera gel were kept for six days at 20 °C, and deferred external color changes were measured as chroma index. In control fruits during storage, a significant decrease in chroma index was noticed, while in treated fruits, the decrease was lower [22]. A substantial difference in fruit colors was observed in peach fruits coated with aloe vera gel, kept in plastic having pores of 5 mm, and cold stored at 1 °C and RH 95% for 30 days with an interval of 10 days as compared to control ones. The coating significantly affected the fruits’ color appearance parameter (hue angle). Aloe vera gel-coated fruits had a greater hue angle throughout storage than uncoated ones. A gradual decrease in hue angle was observed with time. Yet, the coated fruits decrease less than the uncoated ones [55]. From those outcomes, it has been concluded that edible coatings may prevent rapid color change in peach fruits. These outcomes are in accordance with those of [79], who found that aloe vera gel-coated cherry fruits experienced a significantly lower color change in comparison to the control group. “Granada Cultivar” minimally processed peeled peaches were divided into cubes coated with various edible coatings, such as 0.5% xanthan gum, 0.5% Tara gum, 2% alginate, 1% ascorbic acid, 0.25% citric acid, 1% calcium chloride, or 1% glycerol, then subjected to PET packaging and brought to storage for 12 days at 4 ± 1 °C. The color evaluation was carried out on stored peaches. From the experiment, it has been concluded that less alteration in color was observed in Tara-coated treatments in comparison to other treatments and the control group [50].

### 5.2. Firmness

Firmness is the primary physical characteristic of fruit that significantly impacts consumer acceptability. A decrease in firmness was caused by the increased activity of endogenous autolysins, which degrades cell walls [80]. Loss of fruit firmness was found when cell-wall-degrading enzymes like polygalacturonate, pectin methyl esterase, and β-galactosidase decrease cell-to-cell adhesion and the mechanical strength of cell walls as fruit ripens [46]. Those enzymes require oxygen to be functional, and ethylene production enhances their activity in climacteric fruits [77]. It has been reported that edible coatings keep nectarines firmer by slowing down respiration and reducing ethylene production; as a result, the activity of cell-wall-degrading enzymes is minimized. Aloe vera gel-coated nectarine fruit stored for 8 days at a room temperature of 20 ± 1 °C retain greater firmness during ripening than the control group, and the control group shows 40% more softening at the fully ripe stage than coated fruit. The same case was observed in fruits coated with aloe vera gel kept at cold storage at 0 ± 0.5 °C for 3 and 6 weeks at 90 ± 5% RH; retention of fruit firmness was found in coated fruits during ripening, and 41% greater firmness was found at a fully ripe stage as compared to uncoated ones. This was achievable because of lower ethylene production, which results in retarded ripening in “Arctic snow” nectarines coated with aloe vera gel [51].

Moreover, peach fruits coated with methylcellulose and sodium alginate, having storage conditions of 15 °C and 40% RH, retained significantly more firmness during storage in comparison with control fruit [46]. Firmness was significantly higher in the rhubarb-SA (sodium alginate)-coated samples stored for 7 days at ambient temperature (28 ± 1 °C) than in the 1.0% SA (sodium alginate)-coated samples and the control group. Peach fruits with low values of firmness have high weight loss. The higher firmness retained in rhubarb-SA-coated samples was due to excessive antibacterial activity, decelerating intracellular matrix decomposition, and the process and reduction of central vacuole [53]. A significant effect on fruit firmness was observed in aloe vera gel-coated cold-stored peaches for 30 days at 1 °C and 95% RH. A minimum loss of firmness was noticed in aloe vera-coated peaches, and during the late storage period, it was found to be more effective [55]. This outcome was in accordance with [79]. Edible-coated peach fruits with 5 g per liter CS-g-CGA (chitosan chlorogenic acid conjugate) stored at 20℃ for 8 days were found to be more effective in retarding decreases in firmness. In contrast, during the ripening process, the control group’s firmness decreased gradually [52]. 1-Methylcyclopropene (1-MCP)- and aloe arborescens (EC)-coated white peaches cold stored at 1 °C for 28 days showed higher firmness values than 6 kg cm^−2^, which is considered the best for commercial scale. Thus, aloe arborescens, in combination with 1-MCP, has a positive influence on peach fruit firmness by lowering the degradation of cell walls through microbial propagation inhibition and delaying the senescence of fruit. There was no significant decrease in flesh firmness in fruit treated with EC or 1-MCP + EC [56]. Cassava starch, microemulsion based on cassava starch, and commercial wax coatings were applied to whole peach fruits to check the firmness values. Firmness reductions were found in all treatments during storage [81]. These outcomes are in accordance with those of [46]. However, compared to the control sample, they asserted that the coated fruits improved firmness maintenance. Compared to sodium alginate, methylcellulose was more effective at maintaining firmness.

### 5.3. Total Soluble Solids and Titratable Acidity

Total soluble solids (TSS) are the most significant factor through which stone fruits’ eating quality can be determined. TSS measures a fruit’s sweetness and is known to rise throughout ripening as catabolic processes like respiration hydrolyze starch into simple sugars [82]. The breakdown of starch frequently has a higher impact on alteration in TSS values, even though a rise in TSS was found because of postharvest moisture loss resulting from the sugar concentration within fruit [83]. It has been declared that edible coatings reduce the increase in TSS content throughout storage because of repressed respiration [83,84]. The fruit metabolism is decreased by edible coating; as a result, starch breakdown is deferred; consequently, TSS is maintained during storage. Chitosan-chlorogenic acid conjugate (CS-g-CGA)-coated peaches that were stored for 8 days at 20 °C showed that TSS first increased and then decreased at the end of storage; peach fruit coated with 5 g per liter of CS-g-CGA was effective in retarding TSS rise in peach fruit [52], probably as a result of starch hydrolysis and cell wall degradation [85]. Hazrati et al. [55] declared that aloe vera gel-coated fruits that had a storage period of 30 days with 10-day intervals showed that significant changes in TSS were noticed in aloe vera-coated peaches. Aloe vera-coated peaches slightly reduced TSS compared to a control group. These differences may be due to less ethylene production, which leads to later fruit ripening [86]. The values of TSS were found to be 9% higher in the control treatment than in the coated ones. These outcomes are in accordance with those of [51]: aloe vera gel-treated “Arctic snow” nectarine were stored for 8 days at 20 ± 1 °C in the first experiment, while in the second, fruits were subjected to 90 ± 5% RH and 0 ± 0.5 °C for 3 and 6 weeks before they started to ripen at 20 ± 1° C. The first experiment declared that during the starting 3 days, coated and uncoated fruits did not show any significant differences. Later, coated fruits showed lower TSS values than the control during ripening. An increase was found in both coated and uncoated samples, but the rise was more evident in uncoated fruits, whereas the second experiment showed that following 3- and 6-week cold storage, TSS of coated and uncoated samples rose during the period of ripening, but at the ripe stage, uncoated fruits showed 9.4% higher TSS values compared to aloe-coated fruits. White flesh peach fruits “Settembrina” were coated with aloe arborescens (EC), 1-Methylcyclopropene (1-MCP), or EC+1-MCP and stored at 1 °C for 28 days with 7 days of intervals; fruits were then transferred from cold storage to 20 °C and pulled out after 6 days to check the effect of both the room temperature and cold storage on shelf life.

EC+1-MCP significantly reduces TSS values from 7 to 28 days of storage compared with the control group [56]. Cherries fruit coated with almond gum and gum arabic stored for 16 days at 2 °C and 90–95% RH showed that 25% higher TSS was observed in control fruits than coated with gum arabic 18.1% and almond gum 19%. It has been declared that edible coatings reduce ripening and defer starch breakdown [82]. Pizato et al. [50] evaluated “Granada Cultivar” minimally processed peaches peeled off and then divided into cubes. The cubes were then coated with various edible coatings, such as 0.5% xanthan gum, 0.5% Tara gum, 2% alginate, 1% ascorbic acid, 0.25% citric acid, 1% calcium chloride, or 1% glycerol, stored at 4 ± 1 °C for 12 days, and subjected to PET packaging. The control and alginate-coated samples showed an increase in TSS, which may be the result of an accumulation of sugar because moisture loss usually occurs throughout ripening, even in small quantities, whereas Tara gum- and xanthan gum-coated samples show a reduction in TSS and slow down ripening. The fruit TSS may have been leached by immersion in filmogenic solutions [87].

Another crucial aspect of a fruit’s eating quality is titratable acidity (TA). According to Mahfoudhi et al. [82], titratable acidity approximates a solution’s total acidity and frequently declines during postharvest storage because the key substrates for metabolic processes and respiration are organic acids. As fruits mature, their acidity tends to be lower as TSS content rises. It has been reported that edible coatings reduce loss in TA in nectarines [51] and peaches [46] during storage. Ahmed et al. [51] found that aloe-coated nectarine showed 29% significantly higher TA than the control group at the end of storage (stored for 6 weeks at 90 ± 5% RH, 0 ± 0.5 °C, followed by 8 days storage at 60 ± 5% RH, 20 ± 1 °C). Methylcellulose- and sodium alginate-coated peaches stored for 25 days at 15 °C and 40% RH experienced a minimum decrease in TA, particularly methylcellulose-coated peaches, with the maximum being found in the control group, which indicates that peach fruit acid levels reduced slightly faster in the control group than in coated produce. These outcomes are found to be in accordance with [88], which found that peach fruit coated with chitosan reduces acidity at a slower rate. A significant difference in acidity was observed in coated and uncoated fruits throughout the storage period. The control sample showed a maximum acceptable shelf life of 15 days at ambient temperature, which was enhanced to 24 days (60%) by methylcellulose-coated samples and 21 days (40%) by sodium alginate-coated samples [46]. Generally, throughout the storage period, fruit TA declined, which was rapid and more pronounced in the control or uncoated group. Abbas et al. [89] reported that aloe gel-coated fruits had a higher TA (lower pH), which was an indirect sign of lower organic acid metabolism, which was in accordance with [51]. Crisosto et al. [11] discovered that peach titratable acid content reduces by 30% throughout ripening, which agrees with our findings. The TSS and acidity ratio is one of the crucial factors that play a role in determining consumer acceptance of peach fruit. It has also been demonstrated that TSS: TA content is raised throughout storage in fruits 10 days after harvesting; this rise begins but is significantly enhanced between 20 and 30 days at the end. 

## 6. Edible Coatings’ Effect on Physiological Factors of Peach Fruit

### 6.1. Ethylene Production

**Figure 4:**
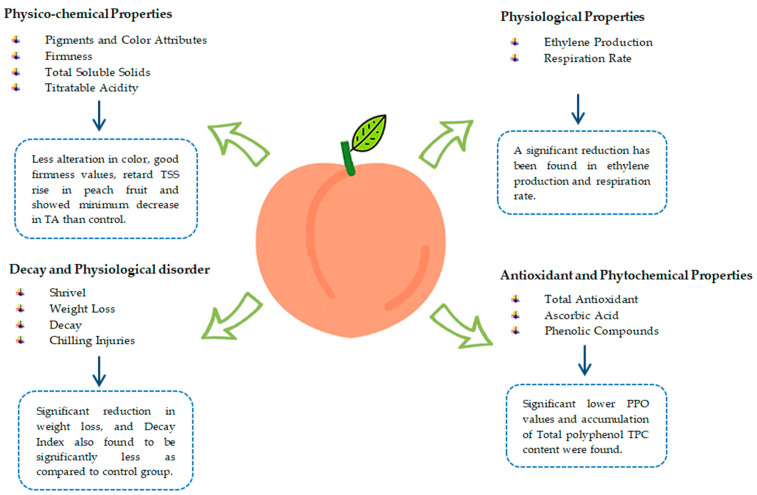
Effect of edible coatings on overall quality of fresh peach fruit.

Ethylene production is usually reduced in coated fruits because of repressed respiration [77]. Aloe-coated peach fruits stored for six days at 85% RH and 20 °C show a 50% reduction in ethylene production during storage [22]. Likewise, a 64% reduction in ethylene production was found in peach fruits coated with glycerol (0.33%), mango seed kernel antioxidant extracts 0.078 g L−1, and mango peel solution 1.09% [90]. Peach gum coating is considered one of the possible alternative treatments to maintain peach fruit functional properties and retard postharvest fruit ripening. Peach fruit treated with different concentrations of peach gum (10%, 5%, and 1%, *v*/*v*) suppressed ethylene production as compared to the control group [54]. Aloe vera gel-coated peach fruits with storage conditions at 1 °C and 95% RH stored for 30 days show less oxygen exchange, so it is likely that ethylene production slows down, which reduces the amount of water lost through the fruit’s peel pores [63]. The ripening of nectarine fruit coated with aloe vera gel kept at room temperature (20 ± 1 °C) for 8 days slowed up until day 6 of ripening, and there was no significant change in ethylene production. Afterward, ethylene production in coated fruits increased but was 62% less than that of the control group on day 8 of ripening. In contrast, the control group had a significant rise in ethylene production. Similarly, fruit coated with aloe vera gel stored for three and six weeks at 90 ± 5% RH and 0 ± 0.5 °C resulted in lower ethylene production during fruit ripening [51]. Figure 4 summarizes the effect of edible coatings on the overall quality of fresh peach fruit.

### 6.2. Respiration Rate

One primary factor contributing to postharvest losses is the respiration rate. Edible films and coatings have the strength to lower the rate of respiration by generating a barrier to carbon dioxide and oxygen by adjusting the internal modified atmosphere [91]. After harvest, the respiration process continues in stone fruit; oxygen is absorbed for utilization in metabolic activities, whereas carbon dioxide and water are released as a by-product [42]. The respiration rate rises continuously during ripening in climacteric stone fruits and becomes maximum at the point where senescence is initiated, which is termed a climacteric peak [44]. The climacteric peak was removed when “Alberta” peaches were kept at 15 °C and 40% RH; these were coated with an edible coating of methylcellulose and sodium alginate, which reduced the rate of respiration by 68% and 62% and allowing them to remain acceptable for 21 days and 24 days, respectively [46]. Applying the rhubarb-SA coating slowed and delayed the peach fruits’ respiration rate. The peak levels were decreased by 11.7% and 16.1% by coatings with 1.0% SA and rhubarb-SA, respectively, and the peak was delayed till day 5. Generally, the respiration rate was effectively decreased by the SA coating combined with rhubarb and retarded the respiratory peak arrival by 1 day in the peach fruit [53]. It has been noticed that a coating made of chitosan or chitosan chlorogenic acid effectively prevented peach fruit respiration rates from rising during storage. The carbon dioxide production rates in peach fruit coated with 5 g per liter CS-g-CGA on days 2, 4, and 6 of storage were 36.5%, 21.9%, and 28.9%, respectively, lower than the control group. It was 16.0%, 10.2%, and 13.0% lower than that of the 5 g per liter CS treatment group, respectively. It has also been concluded that during storage at 20 °C for eight days, the CS-g-CGA coating was more efficient than CS treatment at lowering the gas exchange between the surrounding atmosphere and peach fruit [52]. Nectarine fruit coated with aloe vera gel kept at an ambient condition (20 ± 1 °C) for 8 days shows a significant reduction in the rate of respiration during ripening compared to control fruit; a rapid increase was found in the control fruit respiration rate. Control fruits show a 41% higher respiration rate on day 8 of fruit ripening than aloe vera gel-coated fruits. Similarly, fruit stored at 90 ± 5% RH and 0 ± 0.5 °C for three and six weeks exhibited lower respiration rates during the ripening period than the control group [51].

## 7. Natural Coatings’ Effects on Antioxidant and Phytochemical Properties of Peach Fruit

Stone fruits are reported to have a high nutritional value because of abundant phenolic compounds [92,93]. Phenolic compounds are derived from plant metabolism, considered a class of phytochemicals, and famous for various key properties and biological functions such as antimicrobial activity, pigmentation, protection against UV light, and antioxidant activity. They are the primary antioxidant component of the peach [94,95]. An important subgroup of polyphenols is flavonoids, which contain flavanones, anthocyanins, flavanols, flavones, flavan-3-ols, and isoflavones [96]. A significant rise in both antioxidant capacity and total phenolic content was found in the peel and in the flesh of stone fruit throughout maturation and ripening; compared to the flesh, concentrations are four to five times higher in the peel [97]. As senescence starts in fruit, phenolic compounds reduce because cell structure breaks down [98]. The phenol oxidase and peroxidase activities lower the phenolic content in stone fruits, resulting in lower nutritional value [99]. Total antioxidants and ascorbic acid were significantly lower in edible-coated nectarine fruits with aloe vera gel compared to uncoated ones throughout ripening at cold storage and ambient temperatures. Ascorbic acid levels in coated fruit may have decreased because of the treatment’s general effect on ripening delay, as treatment also reduced respiration rate and ethylene production. The increase in activities of various enzymes such as peroxidase, ascorbic acid oxidase, and cytochrome oxidase may be the cause of the decrease in total antioxidants found in pulp tissues of fruit coated with aloe vera gel, as declared before by [100]. Edible coatings delay the ripening process; as a result, senescence is delayed, and cell structure breakdown is reduced.

Moreover, edible coatings result in decreased respiration and lower oxygen availability in fruits, which are responsible for metabolic activities; consequently, phenol peroxidase and oxidase activities are reduced [46]. Peach fruit treated with 1.0% sodium alginate alone or in combination with rhubarb (rhubarb-SA) stored for 7 days at 28 ± 1 °C showed that a PPO activity was significantly lower in rhubarb-SA-coated fruit throughout storage in comparison with 1.0% SA and the control group. In the control group, the PPO values were 20.8% greater than rhubarb-SA and 14.9% more than 1.0% SA coatings at the end of storage [53]. Postharvest browning in fruits and vegetables is primarily caused by polyphenol oxidase (PPO), as it oxidizes phenolic compounds [101]. PPO activity is linked to the degree of browning on the peach fruit surface [80]. When plant tissue is injured, polyphenols and PPOs, which exist in various cell organelles, react with each other [102], and to stop fruit browning, Chinese herbal medicines can be used as inhibitors to prevent the process that leads to browning. Table 3 summarizes the attitude of fresh peach fruit toward different coating materials.

The edible coating treatment of peach fruits can decrease surface browning by maintaining the structure and integrity of the cell membrane. Therefore, rhubarb-SA-coated peach fruits improved the preservation quality by inhibiting peach PPO activity. Aloe arborescens (EC)-, 1-Methylcyclopropene (1-MCP)- or EC+1MCP-coated settembrina white flesh peach fruits were stored for 28 days at cold storage (1 °C); the fruits were then shifted to room temperature (20 °C) and analyzed to check the combined effect of room temperature and cold storage. It is considered that the total phenolic content (TPC) accumulated because of the EC and EC+1-MCP edible coating, which, as a result, prolonged the shelf and storage life of peach fruits and lowered the incidence of decay. Certain phenolic compounds like flavonoids, phenolic acids, and tannins are found to be directly involved in defense to inhibit pathogen development and make the host tissue stronger. Therefore, during storage and shelf life, it is essential to keep a high-level phenolic compound in fruit [103]. 

## 8. Edible Coatings’ Effect on Decay and Physiological Disorders in Peach Fruit

### 8.1. Shrivel

Shrivel is regarded as one of the most significant physiological disorders affecting stone fruits, resulting in an unpleasant appearance that makes them unsellable [104]. In stone fruits, the development of shrivels is influenced by numerous factors, but it is believed that moisture loss has a huge impact [104,105]. Moisture loss in fruits leads to turgor loss in epidermal cells; as a result, fruits’ overall volume is reduced. The cuticle maintains its surface area, and the elasticity of the cuticle is limited; consequently, there is wrinkled and shriveling development. Additionally, in various cultivars of stone fruits, the occurrence of postharvest shrivel is challenging despite the use of shrivel packaging bags such as high-density polyethylene (HDPE) [104]. It has been stated by Crisosto et al. [106] that nectarine and peaches can shrivel at a minimum of 5% moisture loss rate, and this rate may vary among other cultivars of stone fruits. Edible coatings have the strength to inhibit the development of shriveling in stone fruits. It has been found that caseinate milk protein-coated cherries did not experience shriveling at 4 °C and 80–85% RH when stored for 20 days, whereas the control group experienced high shriveling [107]. To our knowledge, it was concluded that it is the only information that discusses the edible coating ability related to a decrease in shriveling in stone fruits; no study was conducted on edible coating effects on shriveling in peach fruit. Many studies are available on edible coating’s potential to decrease textural losses, peel permeability, and moisture loss [43,108]. As a result, edible coating viability for being used to overcome shrivel development in stone fruit may be favorable. Texture is considered one of the key properties of fruits. Freshness is indicated by appearance. Throughout the ripening process, textural changes occur. Pectin is broken down into protopectin by the pectic enzymes, which lose fruit firmness. The incorporation of texture enhancers into edible coatings has the potential to reduce firmness loss in fruits. The texture of the product is considered a crucial aspect of the acceptability of the product. Stone fruit’s textural loss was found to be linked with shrivel development. When fruit becomes softened, cell-wall-degrading enzymes hydrolyze the pectin, creating a gap in the network of cellulose–hemicellulose [109]. These voids are covered by free water, which binds to cell wall components and decreases the fruit tissues’ overall water movement. In addition, moisture loss may lead to the development of shrivel because of mobile water absence in fruit to keep the hydration beneath the epicarp. Benitez et al. [110] stated that aloe vera-based edible-coated kiwi fruit shows that edible coatings enhanced texture and some other quality parameters. It also enhances the aesthetic value, adds shine to the fruit, and conceals the small scars; even by reducing the loss of moisture, it represses the development of physiological disorders [111]. The coating’s potential to adapt to morphological changes that are peculiar to stone fruit cuticle-like fruit mechanical damage, shrinkage, and shriveling should be excellent.

### 8.2. Weight Loss

Once the fruit is detached from the parent plant, it does not receive more water from the plant, resulting in loss of moisture because of transpiration [112]. Fruit shriveling, softening, and a loss in sellable weight all have been associated with moisture loss and exhibit a parallel trend with the decay rate [113]. Weight loss, which often occurs at postharvest storage, is considered undesirable and often linked to respiration and evaporation of water through epidermal pores [114]. Significant reductions in weight loss were found in edible-coated peaches [55] and nectarines [51,115]. Edible-coated fruits cannot transpire freely because the epicarp of fruits is covered by coating; it also coats the lenticels and stomata and fills the pores and cracks [44]. It has been observed that incorporating lipids into edible coatings enhances their hydrophobicity and has a greater effect on decreasing moisture loss. Peach fruit coated with chitosan CS and chitosan chlorogenic acid conjugate at 5 g per liter CS-g-CGA and stored for 8 days at 20 ℃ showed a significant reduction in weight loss rises compared to uncoated ones. Throughout storage, less weight loss was observed in the peach fruit coated with CS-g-CGA compared to CS coated at the same concentration. Thus, 5 g per liter CS-g-CGA-coated fruits showed the lowest weight loss on day 8 of storage [52].

Aloe vera gel-coated nectarine ’ Arctic Snow’ fruit were kept at an ambient temperature of 20 ± 1 °C for 8 days in the first experiment and left to ripen. It has been observed that a significant weight loss decrease was found in coated fruits throughout ripening. A 65% weight loss decrease was observed at a fully ripe stage in aloe vera coating treatments, while in the second experiment, peach fruits were stored for 3 and 6 weeks at 0 ± 0.5 °C and 90 ± 5% RH before ripening at 20 ± 1 °C. It has been concluded that a significant weight loss decrease was noticed throughout ripening compared to a control group. Fruits coated with aloe vera gel had a 65.3% lower mean weight loss than uncoated ones [51]. The weight loss rate seems to be somewhat repressed in peach gum-treated fruits [54]. Peaches and plum fruits were coated either with aloe arborescens or aloe vera gel and stored for 6 days at 20 °C and left to ripen; a rise in weight loss was observed throughout postharvest storage, having final values of ∼=6.5 and 5.5% in control peaches and plums. However, treated fruits resulted in a significantly lower weight loss. The effect of aloe arborescens is significantly greater than that of aloe vera gel. It has been concluded from these results that compared to aloe vera gel, the gelling properties of aloe arborescens involved in coating formation throughout the fruit surface are enhanced in terms of reduced water vapor permeability [22]. As a result, the hydrophobic properties of aloe arborescens gel would be higher than those of aloe vera gel, resulting in a coating with greater barrier potency [116].

Compared to control treatments, the edible coating of aloe vera gel had a significant effect on the weight loss of peach fruits [55]. Generally, coatings are a combo of fats and polysaccharides that prevent loss of water [117]; because aloe vera gel has a greater polysaccharide content [118], it can be utilized as a non-fatty coating to prevent loss of water in fruits. Weight losses have been found to be significantly decreased in aloe vera gel-coated nectarines [119]. Edible coatings can reduce mass loss as they assist in reducing the loss of water from minimally processed fruits and vegetables [120,121]. “Granada Cultivar” minimally processed peeled peaches were divided into sliced cubes and treated with various edible coatings, such as 0.5% xanthan gum, 0.5% Tara gum, 2% alginate, 1% ascorbic acid, 0.25% citric acid, 1% calcium chloride, or 1% glycerol, stored for 12 days at 4 ± 1 °C, showed that in all treatments, a rise in the mass loss was observed during storage but significant reduction in the mass loss was found in coated peaches as compared to control. Mass loss was a maximum of 34.1% in the control group on day 12 of storage. Tara gum-coated peaches resulted in a minimum mass loss of 7.60%, while among coated samples, xanthan gum-treated peaches showed a maximum mass loss of 10.87%. Thus, regarding mass loss, Tara gum showed the best results [50]. Edible-coated peach fruits with sodium alginate and methylcellulose stored at 40% RH and 15 °C for 24 days showed that in an uncoated control sample after 12 days of storage, mass loss was two folds higher than sodium alginate- and three folds higher than methylcellulose-based edible-coated fruits [46].

### 8.3. Decay

Microbial decay in stone fruits brings about soft rot, brown rot, grey mold rot, and blue or green mold rot caused by *Rhizopus stolonifera*, *Monilinia fructicola*, *Botrytis cinerea*, and *Penicillium expansum*, respectively [83,88,122,123]. In fruits after harvesting, microbial decay is usual because of the high availability of water. Postharvest fruits are susceptible to microbial decay because of the exposure of the huge surface area to an external environment with large amounts of nutrients. Various antimicrobial agents are added to edible coating mixtures to prevent microbial decay by retarding the growth of certain fungi and bacteria and enhancing shelf life. The microbial growth is also controlled by chitosan hydrocolloid, a natural antimicrobial. Numerous microbes get into the food, particularly pathogens that cause foodborne diseases, resulting in major health problems [124]. Antimicrobial coatings are a novel approach for enhancing storage life and increasing microbial safety [125]. The composite coating contains pectin, cinnamon essential oil, and glycerol additive in the peach fruit to boost antioxidant activity and antimicrobial activity [49]. Edible-coated peaches with chitosan chlorogenic acid conjugate 5 g per liter CS-g-CGA stored for 8 days at 20 °C showed less decay index than the chitosan-treated group at the same concentration and storage condition. The 5 g per liter CS-g-CGA edible coating showed a lower DI of about 20.6% compared with a control group and 5 g per liter CS treated, which on the final day of storage exhibited a decay index of 31.2% [52]. Chitosan-based edible coatings have been reported to lower postharvest decay in fruits [126]. Edible-coated peach fruits with sodium alginate stored for 7 days at room temperature 28 ± 1 °C exhibit that decay index results from Penicillium expansum were found to be significantly less during storage than the control group. In contrast, edible-coated rhubarb-SA peaches begin to decay on day 4 of storage, 1.0% SA edible-coated peaches on day 2 of storage, and the control group on day 1 of storage. Rhubarb-SA-coated peaches’ decay index was 65% lower than in the control group at the end of storage. The decay index of rhubarb-SA-coated samples was significantly lower from day 2 than that of the SA-coated group [53]. Stone fruit decay has been reported to be reduced by edible coatings, and when active ingredients are included, the antimicrobial effect is enhanced [42,108]. Sweet cherries coated with an edible coating of guar gum that had been stored for 8 days at 20 °C and 70–75% relative humidity had reduced decay from 43% in the control to 26% in coated cherries; only 13% of the fruit decay were found when an extract of ginseng was added to the coating, which further reduced the incidence of decay [108]. It is broadly reported that chitosan possesses properties of natural antimicrobials [127,128]. Bal et al. [129] reported that plums coated with chitosan stored at 90 ± 5% RH and 0−1 °C for 40 days showed that at the end of storage, the decay rate was reduced to 5.7%, while in uncoated samples, it was 33.1%. It has been reported that in peach fruits, edible coatings of aloe arborescens (EC) alone and in combination with 1-Methylcyclopropene (1-MCP) and aloe arborescens (EC) (1-MCP+EC) induce total phenol content (TPC) accumulation; as a result, chances of decay is lower, and shelf life and storage life is enhanced [103]. The edible coating reduces the chances of fruit decay, enhances the product’s shine, makes it look better, and decreases water loss and mechanical and chilling damage [73]. Edible coatings inhibit microbial contamination [130] and are a barrier against solute movement, moisture, and oxygen [131].

### 8.4. Chilling Injuries

Peaches are regarded as extremely sensitive fruits due to their rapid ripening and deterioration at environmental temperatures. The common method for deferring deterioration in peaches is cold storage preservation, which may lead to chilling injury [132]. The chilling injury has been recognized as a major cause of damage, according to [133,134,135]; it is referred to as a physiological disorder that, according to Lurie and Crisosto [136], is persuaded by low temperatures but not negative ones [18]. According to these authors, chilling injury usually occurs when the storage temperature ranges from 22 °C to 7.6 °C, resulting in loss of flavor, dry texture, and taunting floury pulp. Numerous studies have shown that chilling injury has been viewed as the peach industry’s major issue in recent years [137]. Symptoms of chilling injury are more likely to appear in fruit stored at a temperature ranging from 2 °C to 7 °C. Instead, products are stored at 0 °C or a lower temperature that is not above freezing [106]. Hence, to avoid such postharvest disorders during the entire handling process, keeping the product at suitable temperatures is crucial. The susceptibility of stone fruit cultivars to chilling injury symptoms varies, with peach cultivars being more susceptible than nectarine cultivars [18]. The development of this phenomenon has been combatted or slowed down by various treatments and methods, always to preserve the organoleptic features of the peach [132]. It has been reported that cold storage also results in chilling injury symptoms like internal breakdown, wooliness, flesh translucency, and flesh bleeding [44,77].

Additionally, nectarines are easily subject to physiological disorders during low-temperature storage, like woolliness and chilling injury [138]. Usually, chilling injury results in nectarine and peaches at 8 °C. Chilling injury’s visible symptoms are lack of juiciness, flavor loss, mealiness or wooliness, and internal pulp browning [54]. In chilling injury, long-term exposure to carbon dioxide results in off-flavor and health risks of conventional fungicides. For future research, edible coatings have been of great interest [139]. There is a dearth of information regarding the effects of edible coatings on the symptoms of chilling injury in peach fruits. Navarro-Tarazaga et al. [140] stated that in plums, composite coating of hydroxypropyl methylcellulose lowered flesh bleeding compared to a control group when stored for 8 weeks at 1 °C and 85% RH. However, there is no other evidence to our knowledge that edible coatings affected the symptoms of chilling injury in peach fruit. From several studies, it has been concluded that edible coatings have a positive influence on chilling injury symptoms during cold storage in postharvest horticultural products. Various edible coatings have been developed and were found to be excellent, such as galactomannan-carnauba wax, resulting in guavas resistant to chilling injury stored at 11 °C [141], and in orange fruits, aloe vera gel in combination with salicylic acid reduces symptoms of chilling injury at 4 °C [142].

## 9. Conclusions and Prospects

Edible coatings, an eco-friendly alternative to plastic packaging, are gaining traction in the food processing sector. These coatings, often polysaccharide-based, create a protective layer on fresh produce, extending shelf life by inhibiting oxygen, carbon dioxide, and water penetration. The addition of lipids enhances moisture barrier properties, while antioxidants and antimicrobial agents further improve functionality. This technology shows promise in significantly reducing postharvest losses and preserving the quality and safety of food, particularly in extending the shelf life of peach fruit. Edible coatings, a “green technology”, show significant potential in enhancing the postharvest management of peach fruit. While laboratory studies indicate positive impacts, there is a crucial gap in understanding how these coatings perform on a commercial scale. Current research emphasizes the need for realistic, large-scale trials to evaluate the commercial viability of edible-coated peaches. Bridging this gap is essential to unlock the full potential of these coatings and address the intense need for natural, sustainable, and cost-effective postharvest preservation methods in the peach industry. Incorporating active ingredients in edible coatings alters their properties, necessitating further research on their impact on shelf life and mechanical and functional aspects. Fruit–coating interactions require attention to avoid undesirable compounds. Key aims include emphasizing nanotechnology, exploring cost-effective base alternatives, and developing labor-efficient methods. Future research should prioritize tailor-made edible coatings that are versatile across products, enhancing functional properties and shelf life. Addressing gaps in available information, future research seeks new, valuable coating applications with improved sensory and functional attributes. Future experiments ought to concentrate primarily on the degree of technological readiness of edible coating applications in peach fruit. When evaluating the performance of edible coatings on a commercial scale, the effectiveness of various application methods should be taken into account, as well as handling practices, costs, and the sustainability of edible coating as alternative postharvest technology. The worldwide peach fruit industry may benefit from adopting this scientific tool for edible coating technology.

## Figures and Tables

**Table 1:** Fresh peach fruit’s major quality attributes for marketability.

Quality Aspect	Primary Concern
Texture (mouth feel)	Fibrousness/toughness
	Juiciness
	Crispness
	Softness/firmness
Appearance (visual)	Defects
	Gloss
	Size
	Form and shape
	Color (uniformity, intensity)
Nutritional value	Minerals
	Vitamins
Flavor (aroma, taste)	Astringency
	Acidity
	Bitterness
	Sweetness
	Volatile compounds
Safety	Chemical contaminants
	Toxic substances
	Microbial contamination

**Table 2:** Effect of various edible coatings on peach fruit quality.

Fruit	Edible Coating	Formulations	Storage Condition	Findings	References
Peach (‘Alberta’)	Methylcellulose (MC) and Alginate (A)	MC 3%, A 2%	15 °C and 40% RH, stored for 21 days (A) and 24 days (MC)	Moisture loss, changes in quality parameters, and respiration rate were much lower in coated peaches. MC-coated fruit shows (68%) a high effect regarding respiration than A (62%).	[46]
Peach (Persica vulgaris Miller.)	Wax and Carboxymethylcellulose (CMC)		25 °C and 75% RH, stored for 12 days	Reduces respiration, weight loss, and moisture barrier and partially inhibits PPO activity.	[47]
Nectarines	Carnauba Wax			Retards fungal and enhances brightness and firmness of fruit, and no spore germination of R. stolonifera and M. fructicola.	[48]
Peach (‘Jefferson’)	Pectin and Cinnamon Essential Oil (EO)		5 °C and 90% RH, stored for 15 days	Enhances antioxidant and antimicrobial activity. Decreases microbial growth. Retards color, taste, and flavor changes.	[49]
Peach (‘RedHeaven’)	Aloe Vera or Aloe Arborescens gels		20 °C and 85% RH, stored for 6 days	Retards ethylene production and weight loss. A significant delay was found in color change and ripening index.	[22]
Peach (Granada cultivar)	Xanthan Gum, TaraGum, Sodium Alginate	Xanthan gum (0.5% *w*/*v*), tara gum (0.5% *w*/*v*), alginate (2% *w*/*v*)	4 ± 1 °C, stored for 12 days	Tara gum showed the best result and led to firmness maintenance, mass loss reduction, lower mold and yeast growth, and lower color alteration.	[50]
Nectarine (‘Arctic Snow’)	Aloe Vera Gel	Aloe vera gel dried powder (200:1)	20 ± 1 °C and 60 ± 5% RH, stored for 8 days. In separate trials, fruits were kept at 0 ± 0.5 °C and 90 ± 5% RH for 3 and 6 weeks before ripening at 20 ± 1 °C	Reduction was found in respiration rate, production of ethylene, electrolyte leakage, weight loss, and maintaining firmness.	[51]
Peach	Chitosan (CS), Chitosan Chlorogenic Acid Conjugate CS-g-CGA		20 °C, stored for 8 days	CS-g-CGA maintained soluble solid contents, firmness, titratable acidity, and L-ascorbic acid contents well. It also prevented decay index, respiration rate, and weight loss increase.	[52]
Peach	Sodium Alginate (SA), Rhubarb	1% sodium alginate and rhubarb-SA	28 ± 1 °C and 90% RH, stored for 7 days	Reduction was found in respiration rate, weight loss, PPO Activity and MDA content. It also has good anti-fungal activity.	[53]
Peach (‘Jinxiu’)	Peach Gum	Peach gum 1%, 5% and 10% *v*/*v*	8 °C, stored for 25 days	Fruit softening, ethylene production, and reduction in sorbitol are repressed. Weight loss is also reduced.	[54]
Peach	Aloe Vera Gel	A. gel diluted with distilled water (1:3)	1 °C and 95% RH, stored for 30 days	Reduction in total soluble solids (TSS), weight loss, titratable acidity (TA) color change. Increased visual properties have more favorable texture and flavor.	[55]
White Peach Fruit (‘Settembrina’)	Aloe Arborescens (EC), 1-Methycyclopropene (1-MCP)	EC 40% (*v*/*w*), 1-MCP 0.14%, EC 40% (*v*/*w*) + 1-MCP 0.14%	Stored at 1 °C for 28 days, whereas in second trial, fruits were transferred at 20 °C for six days	Retards ripening and maintains flesh firmness, color, and weight. Decreases transpiration and respiration.	[56]

**Table 3:** Attitude of fresh peach fruit toward different coating materials.

Effect of Edible Coatings on Physico-Chemical Properties of Peach			
Quality Parameter	Coating Material	Effect of Coating	References
Pigment and color attributes	Aloe arborescens and aloe vera gel.	Deferred external color changes. A lower decrease in chroma index.	[22]
	Aloe vera gel	Greater hue angle. Gradual decrease in hue angle. Yet, less decrease in comparison to coated ones.	[55]
	Tara gum	Less alteration in color as compared to control group.	[50]
Firmness	Methylcellulose and sodium alginate	Retained significantly more firmness during storage in comparison with control fruit.	[46]
	Rhubarb-SA (sodium alginate)	Showed significantly higher firmness than control group.	[53]
	Aloe vera gel	Minimum firmness loss was observed at later storage period.	[55]
	CS-g-CGA (chitosan chlorogenic acid conjugate)	Retard decrease in firmness.	[52]
	1-Methylcyclopropene (1-MCP) and aloe arborescens (EC)	Showed good firmness values.	[56]
	Cassava starch, microemulsion based on cassava starch, and commercial wax coating	Reduction in firmness was observed in all treatments.	[81]
Total soluble solids	CS-g-CGA (chitosan chlorogenic acid conjugate)	Retarded TSS rise in peach fruit	[52]
	Aloe vera gel	Aloe vera-coated peaches show a slight reduction in TSS compared to a control group.	[55]
	Aloe arborescens (EC), 1-Methylcyclopropene (1-MCP), or EC+1-MCP	A significant reduction in TSS values in comparison with the control group	[56]
Titratable acidity	Tara gum and xanthan gum	Showed a reduction in TSS and slowed down ripening.	[87]
	Methylcellulose and sodium alginate	Experienced a minimum decrease in TA compared to control group.	[46]
	Chitosan	Reduces acidity at slower rate.	[88]
Effect of edible coatings on physiological factors of Peach Fruit.			
Ethylene production	Aloe vera gel	Reduction in ethylene production during storage.	[22,51]
	Glycerol, mango seed kernel antioxidant extracts, and mango peel solution	64% reduction in ethylene production was found.	[90]
Respiration rate	Methylcellulose and sodium alginate	Reduced the rate of respiration by 68% and 62%, respectively.	[46]
	Chitosan or chitosan chlorogenic acid	Prevented peach fruit respiration rates from rising during storage.	[52]
Effect of edible coatings on antioxidant and phytochemical properties of peach fruit.			
Antioxidant and phytochemical properties	Sodium alginate alone or in combination with rhubarb (rhubarb-SA)	Showed significant lower PPO values in rhubarb-SA-coated fruit throughout storage in comparison with 1.0% SA and control group. The PPO values were found to be 20.8% greater in control group than with rhubarb-SA 14.9% and SA coatings 1.0%.	[53]
	Aloe arborescens (EC) and 1-Methylcyclopropene (1-MCP), or EC+1MCP	Accumulation of total phenol content (TPC), which prolonged the shelf life and lowered the decay incidence.	[103]
Effect of edible coatings on decay index and physiological disorders of peach fruit.			
Weight loss	Chitosan CS and chitosan chlorogenic acid conjugate	Significant reduction in weight loss during storage.	[52]
	Aloe vera gel	Throughout ripening, a significant weight loss decrease was noticed. Aloe vera gel had a 65.3% lower mean weight loss than uncoated ones.	[51]
	Tara gum	Tara gum-coated peaches resulted in a minimum mass loss of 7.60%. Thus, in terms of mass loss, Tara gum showed the best results.	[50]
Decay index	Chitosan chlorogenic acid conjugate (CS-g-CGA)	Showed lower decay index of about 20.6% in comparison with a control group.	[52]
	Sodium alginate	Decay index was found to be significantly less as compared to control group.	[53]
